# Targeting Orphan G Protein-Coupled Receptor 17 with T0 Ligand Impairs Glioblastoma Growth

**DOI:** 10.3390/cancers13153773

**Published:** 2021-07-27

**Authors:** Phuong Doan, Phung Nguyen, Akshaya Murugesan, Kumar Subramanian, Saravanan Konda Mani, Vignesh Kalimuthu, Bobin George Abraham, Brett W. Stringer, Kadalmani Balamuthu, Olli Yli-Harja, Meenakshisundaram Kandhavelu

**Affiliations:** 1 Molecular Signaling Lab, Faculty of Medicine and Health Technology, Tampere University, P.O. Box 553, 33101 Tampere, Finland; phuong.doan@tuni.fi (P.D.); phunghatien.nguyen@tuni.fi (P.N.); akshaya.murugesan@tuni.fi (A.M.); kumar.subramanian@tuni.fi (K.S.); 2 BioMediTech Institute and Faculty of Medicine and Health Technology, Tampere University, Arvo Ylpön Katu 34, 33520 Tampere, Finland; 3 Department of Biotechnology, Lady Doak College, Thallakulam, Madurai 625002, India; 4 Bharath Institute of Higher Education and Research, Chennai 600073, India; saravananbioinform@gmail.com; 5 Department of Animal Science, Bharathidasan University, Tiruchirappalli 620024, India; ksvignesh738@gmail.com (V.K.); kadalmani@bdu.ac.in (K.B.); 6 Faculty of Medicine and Health Technology, Tampere University, P.O. Box 553, 33101 Tampere, Finland; bobin.george.abraham@tuni.fi; 7 College of Medicine and Public Health, Flinders University, Sturt Rd., Bedford Park, SA 5042, Australia; Brett.W.Stringer@gmail.com; 8 Computational Systems Biology Group, Faculty of Medicine and Health Technology, Tampere University, P.O. Box 553, 33101 Tampere, Finland; olli.yli-harja@tuni.fi; 9 Institute for Systems Biology, 401 Terry Ave N, Seattle, WA 98109, USA

**Keywords:** glioblastoma, GPR17-targeted drug, mode of action, cell death, toxicity, blood–brain barrier, in vivo

## Abstract

**Simple Summary:**

Glioblastoma multiforme (GBM), or glioblastoma chemotherapy, has one of the poorest improvements across all types of cancers. Despite the different rationales explored in targeted therapy for taming the GBM aggressiveness, its phenotypic plasticity, drug toxicity, and adaptive resistance mechanisms pose many challenges in finding an effective cure. Our manuscript reports the expression and prognostic role of orphan receptor GPR17 in glioma, the molecular mechanism of action of the novel ligand of GPR17, and provides evidence how the T0 agonist promotes glioblastoma cell death through modulation of the MAPK/ERK, PI3K–Akt, STAT, and NF-κB pathways. The highlights are as follows: GPR17 expression is associated with greater survival for both low-grade glioma (LGG) and GBM; GA-T0, a potent GPR17 receptor agonist, causes significant GBM cell death and apoptosis; GPR17 signaling promotes cell cycle arrest at the G1 phase in GBM cells; key genes are modulated in the signaling pathways that inhibit GBM cell proliferation; and GA-T0 crosses the blood–brain barrier and reduces tumor volume.

**Abstract:**

Glioblastoma, an invasive high-grade brain cancer, exhibits numerous treatment challenges. Amongst the current therapies, targeting functional receptors and active signaling pathways were found to be a potential approach for treating GBM. We exploited the role of endogenous expression of GPR17, a G protein-coupled receptor (GPCR), with agonist GA-T0 in the survival and treatment of GBM. RNA sequencing was performed to understand the association of GPR17 expression with LGG and GBM. RT-PCR and immunoblotting were performed to confirm the endogenous expression of GPR17 mRNA and its encoded protein. Biological functions of GPR17 in the GBM cells was assessed by in vitro analysis. HPLC and histopathology in wild mice and an acute-toxicity analysis in a patient-derived xenograft model were performed to understand the clinical implication of GA-T0 targeting GPR17. We observed the upregulation of GPR17 in association with improved survival of LGG and GBM, confirming it as a predictive biomarker. GA-T0-stimulated GPR17 leads to the inhibition of cyclic AMP and calcium flux. GPR17 signaling activation enhances cytotoxicity against GBM cells and, in patient tissue-derived mesenchymal subtype GBM cells, induces apoptosis and prevents proliferation by stoppage of the cell cycle at the G1 phase. Modulation of the key genes involved in DNA damage, cell cycle arrest, and in several signaling pathways, including MAPK/ERK, PI3K–Akt, STAT, and NF-κB, prevents tumor regression. In vivo activation of GPR17 by GA-T0 reduces the tumor volume, uncovering the potential of GA-T0–GPR17 as a targeted therapy for GBM treatment. Conclusion: Our analysis suggests that GA-T0 targeting the GPR17 receptor presents a novel therapy for treating glioblastoma.

## 1. Introduction

Glioblastoma (GBM) is an aggressive neoplastic tumor, clinically featured by infiltrative high-grade glioma cells into the brain parenchyma with poor response to treatment [1]. Patients have a median survival time of less than 1.5 years, despite surgery, radiation, and chemotherapy [2]. The dynamic microenvironment of GBM is primarily due to the propensity of neoplastic cells to migrate from the primary tumor mass into nearby tissues [3]. GBM is enriched with unique phenotypic properties, including self-renewal [4,5], hypoxic adaptations [6], genetic lesions [7], and resistance to radiation and chemotherapeutic agents [8]. In addition, gene expression analysis of patient tumor tissue has identified phenotypically distinct molecular subtypes of GBM [9,10,11], based on the chaotic oscillation of tumor cells [12]. Although multiple subtypes can co-exist in the affected individual, transcriptional dominance defines the incidence of the specific tumor type [13]. The complex cellular and molecular heterogeneity in GBM exists both between patients and within the individual’s tumor. All these features, along with the genetic, transcriptional, and functional variation inherent to GBM, contribute to treatment failure, and effective therapeutic strategies remain obscure [14,15]. Therefore, designing new approaches to identify promising drugs or targets for GBM treatment is pivotal, especially targeting the signaling receptors envisaged to subvert cellular communication [16] for disease progression and recurrence.

G protein-coupled receptors (GPCRs), a large superfamily of signaling receptor molecules, have been considered an interesting pharmacological target for numerous pathological conditions [17]. They function explicitly by their accessible “druggable receptor” sites at the cell surface [18]. The iterative structure–function relationship revealed through advanced X-ray crystallographic methods has lifted the structural veil of the receptor, signifying a new era of GPCR-based drug discovery. GPCR-targeted drugs are rapidly emerging for cancer treatment and at least 23 GPCR-targeted agents are in clinical trials, representing its therapeutic interest [19].

An orphan GPCR receptor, GPR17, is an enigmatic receptor that respond to both endogenous purinergic and cysteinyl-leukotriene (CysLT) [20,21] and to synthetic ligands, such as pranlukast and MDL29951, which (ant)agonize, respectively [20,22,23]. GPR17 is a sensor of demyelinated tissues caused by inflammatory responses and crucially promotes the differentiation of the precursor oligodendrocyte into mature cells at the site of plaques or lesions [24,25]. GPR17 clustering is associated with the overexpression of transcription factors such as Olig1 and Olig2 in pediatric diffuse midline glioma (pDMG), with the aborted differentiation of the oligodendrocytic lineage of the cells [26]. A similar hypothesis reflects the role of GPR17 as a candidate agonist gene in decreasing the number of neurospheres in primary murine GBM cells [27]. The limited insight [28,29] into GPR17 signaling in GBM and its tumor microenvironment prompted us to investigate the mechanism of GPR17 signaling activation, the downstream effects, its role in cell death and therapeutic applications in GBM treatment.

## 2. Results

### 2.1. GPR17 as a Biomarker for LGG and GBM

**Figure 1:**
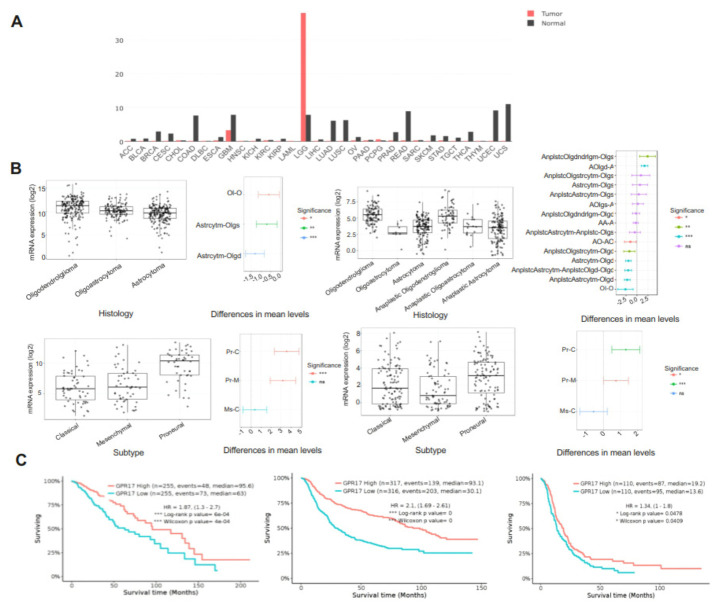
GPR17 as a biomarker in histological subtypes of glioma. (**A**) GPR17 expression profile across all tumor samples and paired normal tissues. (**B**) GPR17 expression in histological subtypes of glioma. (**C**) Overall survival associated with GPR17 expression in the TCGA LGG dataset, CGGA primary glioma dataset, and CGGA primary GBM dataset; survival time is represented in months.

We investigated GPR17 expression from publicly available RNAseq gene expression cancer datasets using the GEPIA portal. There is conspicuous expression of GPR17 mRNA in LGG and in GBM, although in the latter cases the expression was less than the level detected in matched normal tissue (Figure 1A and Supplementary Figure S1). Consistent with the known expression of GPR17 in oligodendrocyte precursor cells, elevated GPR17 expression in LGG was highest in the histological subtypes with an immature oligodendroglial component (Figure 1B). Likewise, expression of GPR17 was greatest in the proneural subtype GBM (Figure 1B), which is believed to arise from oligodendroglial precursor cells or have an oligodendroglial phenotype. A univariate analysis of the association between GPR17 expression and overall survival in LGG and GBM demonstrated GPR17 expression to be a strong predictive biomarker of improved survival in both the TCGA and CGGA datasets (*p* = 6 × 10^−4^ and 0, respectively) (Figure 1C). GPR17 expression was also associated with improved survival in GBM alone (*p* = 0.0478) in the CGGA dataset, although not in the TCGA dataset. Thus, these RNAseq data revealed an association of GPR17 expression with both LGG and GBM and showed GPR17 to be a strong positive predictive biomarker in LGG and possibly also in GBM.

### 2.2. GA-T0 Activates GPR17 Signaling in GBM Cell Lines

**Figure 2:**
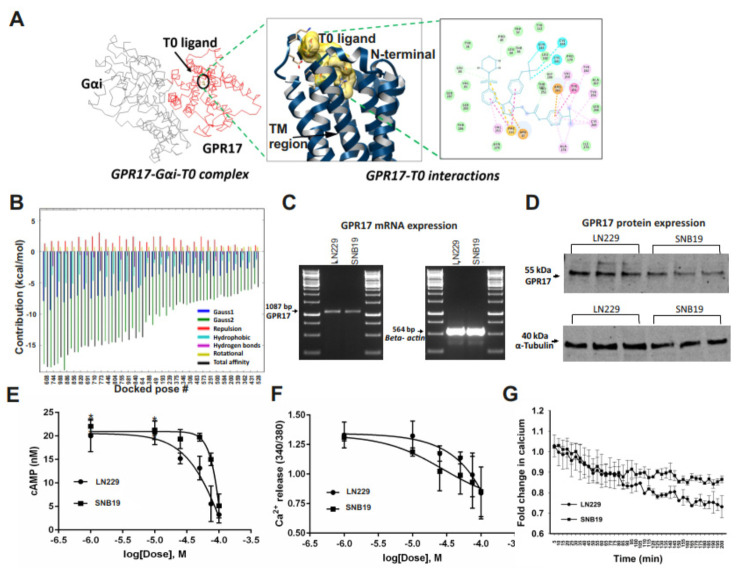
**GA–T0 as a potential GPR17 agonist in GBM cells.** (**A**) GPR17–GαI complex obtained from the protein–protein docking where GPR17 is colored red, GαI is grey, and the GA-T0 complex in the black circle. The portion of GPR17–GA-T0 is magnified in the 2-dimensional and 3-dimensional interaction figure. (**B**) Docked structure of the GPR17–GαI–GA-T0 complex ranked according to binding energy from different interactions, including hydrogen bonds, hydrophobic interaction, ion pair interaction, aromatic interaction, and cation pi interaction. (**C**) RT-PCR analysis of the GPR17 receptor expression in LN229 and SNB19 cells, using β-actin as a constitutive control. (**D**) Immunoblot analysis of GPR17 suppressing cell proliferation in LN229 and SNB19 cells with α-tubulin as the loading control. (**E**) cAMP level (nM) and (**F**) ratiometric (340/380 nm) analysis of Ca2+ release in the SNB19 and LN229 cell lines on treatment with GA-T0. (**G**) Fold change in the Ca^2+^ level over the time (min) in GBM cells. Data are representative of at least three independent experiments (*n* = 6) using t-test analysis. (**E–G**) The results are presented as the mean values ± SEM of six experiments. Significant data are denoted by asterisks (*, *p* < 0.05).

To investigate GPR17 signaling in GBM using a potent ligand, protein–protein-agonist blind docking experiments were performed. Consistent with our previous work on GA-T0 as a novel agonist of GPR17 [30], GPR17–GαI was complexed with GA-T0 (Figure 2A). A two-dimensional protein–ligand interaction plot was generated, which revealed that GA-T0 formed 33 interactions with the amino acid residues of the GPR17 receptor, better than the previously known agonist, MDL 29,951, which exhibits 22 interactions. GA-T0 also exhibited a better binding energy (−18.5 Kcal/mol) than MDL 29,951 (−13.4 Kcal/mol), and 30.76 Å, 59.81 Å, and 8.31 Å are the binding site coordinates (Figure 2B, Supplementary Figure S2). We next investigated GA-T0-mediated GPR17 signaling activation in the GBM cell lines LN229 and SNB19. Endogenous expression of GPR17 mRNA and protein in both cell lines was confirmed by real-time PCR and immunoblotting using GPR17-specific primers and antibodies (Figure 2C,D, Supplementary Figure S4). We further addressed the downstream signaling activation of GPR17 by the GA-T0 agonist in GBM cells by quantifying the level of the secondary messenger cAMP. The GA-T0–GPR17–Gαi interaction regulates the decrease in forskolin-stimulated intracellular cAMP by reducing the adenylyl cyclase activity (Figure 2E), with an EC_50_ of 76.64 µM and 42.05 µM for SNB19 and LN229, respectively. Simultaneously, GA-T0 shows inverse agonism for the calcium level in GBM cells, suggesting Gαq-independent signaling activation of GPR17 in a dose- (Figure 2F) and time-dependent manner, with an EC_50_ of 19.64 µM and 47.33 µM for SNB19 and LN229, respectively (Figure 2G).

### 2.3. GPR17 as a Target for Inhibiting GBM Cell Proliferation

**Figure 3:**
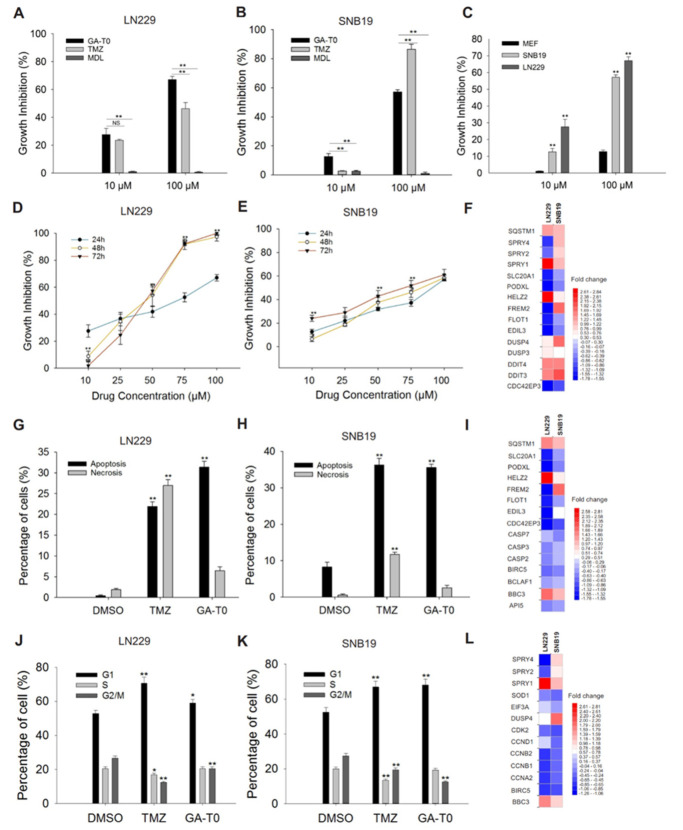
**Effect of GA-T0 on glioblastoma cell growth, apoptosis, and the cell cycle.** Percentage of cell growth inhibition at 10 μM and 100 µM concentrations of GA-T0, MDL 29,951, and TMZ in (**A**) LN229, (**B**) SNB19, and (**C**) non-tumor cells (MEF). Dose- and time-dependent effect of GA-T0 on (**D**) LN229 and (**E**) SNB19 at 10, 25, 50, 75, and 100 µM for 24 h, 48 h, and 72 h, respectively. Top DEGs associated with (**F**) DNA damage. Percentage of apoptosis and necrosis in (**G**) LN229 and (**H**) SNB19 on treatment with DMSO, TMZ, and GA-T0 and (**I**) DEGs involved in apoptosis. Percentage of cells in different stages of the cell cycle (G1, S, and G2/M phase) in (**J**) LN229 and (**K**) SNB19 on treatment with DMSO, TMZ, and GA-T0 and (**L**) DEGs associated with cell cycle arrest. Data are the mean ± SD of six experiments using t-test analysis. Non-significant data are denoted by NS and significant data by asterisks (*, *p* < 0.05 and **, *p* < 0.01).

To investigate the signaling effect of GPR17 on the proliferation of GBM cells, the percentage of cell growth inhibition was evaluated. At 10 µM, GA-T0 caused significantly greater inhibition of proliferation of LN229 and SNB19 cells than did MDL 29,951 and TMZ. At 100 µM, the effect of GA-T0 on GBM cell proliferation was greater still, and again significantly greater than MDL 29,951, although not as great as TMZ against LN229 cells. Interestingly, MDL 29,951 has a negligible cytotoxic effect (1% to 2%) on both GBM cell lines (Figure 3A,B). In contrast to its effect on GBM cells, GA-T0 had a much smaller effect on the proliferation of normal cells (mouse embryonic fibroblasts). Even at a 100 µM concentration, GA-T0 inhibited the proliferation of MEFs < 15% (Figure 3C). Thus, GA-T0 was found to be a unique agonist inducing GPR17-mediated inhibition of GBM cell proliferation.

Treatment with GA-T0 also strongly reduced GBM cell proliferation in a time-dependent as well as dose-dependent manner, reaching 100% for LN229 cells at 48 h and 60% for SNB19 cells. The IC_50_ concentrations for LN229 (Figure 3D) were observed to be 86 µM, 44 µM, and 43 µM, and for SNB19 were 98 µM, 95 µM, and 95 µM (Figure 3E) at 24, 48, and 72 h of GA-T0 treatment, respectively, suggesting the cytotoxicity increased over time.

DNA damage can impinge on the proliferation of tumor cells and thus hampers the progression of the disease. To directly assess the genes involved in DNA damage by GA-T0 on GBM cells, we performed total RNA expression analysis of GA-T0-treated LN229 and SNB19 cells. We found upregulation of *DDIT3* [31], *DDIT4* [32,33], and *SQSTM1* [34] in both GBM cell lines, confirming its promising role in DNA damage (Figure 3F). 

### 2.4. Apoptosis-Mediated Cell Death Induced by GA-T0

The effect of apoptosis on GBM cells was identified by detecting the externalization of phosphatidylserine (PS) to the outer plasma membrane. GA-T0 shifted nearly 32% of the LN229 cells from viable cells to apoptotic cells (Figure 3G), and 35% of the SNB19 cells (Figure 3H). A similar pattern was observed following TMZ treatment, with 21.9% apoptotic cells for LN229 and 35% for SNB19 cells. In contrast, the percentage of necrotic cells was 7% and 4% for GA-T0 while 27% and 12% for TMZ in the LN229 and SNB19 cell lines, respectively.

We additionally validated the genes involved in apoptosis-mediated cell death through gene expression profiling. Apoptotic inhibitor genes, such as survivin, *BIRC5* [35], and *API5* [36], were downregulated in both GBM cell lines, with the upregulation of the pro-apoptotic gene, *BBC3*, in SNB19 cells, whose expression increases in response to diverse apoptotic stimuli [37] (Figure 3I). *BCLAF1*, an anti-apoptotic Bcl-2 family member, was found to be downregulated in LN229 cells, suggesting a role in GPR17-mediated apoptosis at physiological levels [38,39]. The downregulation of *CASP2* [40] and *CASP3* [41,42] in LN229 cells and *CASP7* [41] in SNB19 cells also supports the likelihood that GPR17 activates a caspase-independent mechanism of apoptosis.

### 2.5. GA-T0 Promoted Cell Cycle Arrest at the G1 Phase

To determine whether GPR17 signaling promotes cell cycle arrest, the percentage of cells in each phase of the cell cycle was analyzed. Following GA-T0 treatment, we observed significant arrest of GBM cells in the G1 phase, with a concomitant decrease in the percentage of cells in the S and G2/M phase at 24 h (*p* < 0.01 for LN229 and *p* < 0.05 for SNB19). As shown in Figure 3J, GA-T0-treated LN229 cells were found to have 59% arrest at the G1 phase, which increased to 68.2% for SNB19 cells (Figure 3K). Similarly, TMZ also arrested GBM cells in the G1 phase, with 70.7% for LN229 cells and 67% for SNB19 cells. 

These results were correlated with the differential expression of genes involved in the cell cycle. Notably, downregulation of the *CDK2* gene was observed in both GBM cell lines, suggesting a pivotal role in cell cycle regulation [43]. This perturbates the p53 signaling pathway, which, in turn, activates the p21 pathway by downregulating several cyclins [44], such as *cyclin E2* in LN229 and *cyclin D1* and *cyclin D3* in SNB19 cells (Supplementary Tables S1 and S2). The downregulation of cyclin-specific genes, such as *CCNE1, CCND1* (restricted to SNB19), and *CCND3*, a regulatory subunit of *CDK2*, further suggests potential defects in the transition of the G1 to S phase of the cell cycle. We also noted downregulation of cyclin A2, cyclin B1, and cyclin B2, encoded by *CCNA2, CCNB1*, and *CCNB2*, respectively, which potentially prevented the transition of cells from the G2 to M phase (Figure 3L). These results indicated the potential role of the GPR17 signals in maintaining efficient cell cycle progression by inducing cell cycle arrest at the G1 phase. 

### 2.6. Effect of GA-T0 Mediated GPR17 Activation on Signal Transduction Pathways

#### 2.6.1. PI3K–Akt Pathway

**Figure 4:**
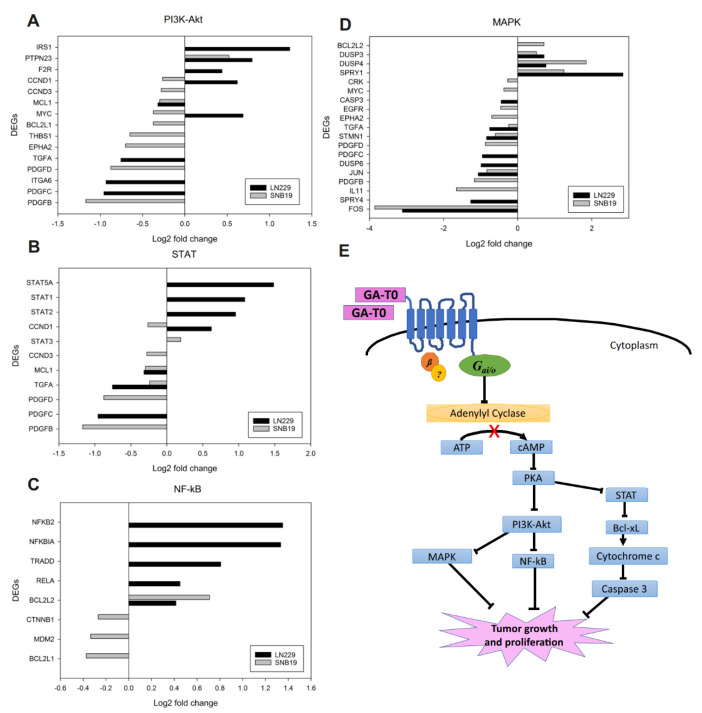
**Regulation of signal transduction pathways upon GA–T0 treatment**. (**A**) Key DEGs associated with the PI3K–Akt, (**B**) STAT, (**C**), NF-κB, and (**D**) and MAPK pathways. (**E**) Schematic overview of signal transduction pathway modulation upon the binding of GA-T0 on the GPR17 receptor in GBM cells.

*MCL1*, an anti-apoptotic Bcl-2 family gene that promotes survival of glioma cells by preventing apoptosis [45], was found to be downregulated in both GBM cell lines. *MCL1* inhibition in the PI3K–Akt pathway intriguingly supported our study on the role of GPR17 in arresting the cell cycle at the G1 phase, thus reducing cellular proliferation and in turn increasing senescence and apoptosis [46]. Therefore, silencing *MCL1* by GA-T0 also could target CREB protein [47], a downstream transcription factors of the PI3K/Akt signaling pathway, which is highly regulated in most cancers. Another notable gene, Protein Tyrosine Phosphatase N23, *PTPN23* whose downregulation is correlated with poor survival in breast cancer, was observed to be upregulated in GA-T0 treated GBM cell lines. Also, activation of PI3K/Akt is observed in prostate cancer disease progression upon the loss of PTP1B [48], a precedent gene of *PTPN23* (Figure 4A). These observations suggest GPR17 targeting of the PI3K–Akt pathway, thus preventing GBM proliferation.

#### 2.6.2. STAT Pathway

Persistent activation of the STAT pathway contributes to tumor proliferation and survival in the microenvironment and promotes tumor growth [49,50]. The inhibition of *MCL1* activation in the STAT pathway revealed the potential role of GPR17 as a signal transducer in GBM cell lines. The Gαi-mediated reduction in the level of cAMP by GA-T0 (Figure 2F and Figure 4E) supports the fact that the reduced binding of the cAMP response element (CRE) to the promoter region of *MCL1* downregulates its expression [51]. As noted earlier, induced apoptosis by the drug could also transcriptionally downregulate *MCL1* [52]. Likewise, TGFα downregulation, a mitogenic protein, incriminates the agonistic role of GA-T0 in forming autocrine looping, supporting the antiproliferation of human glioma [53] (Figure 4B).

#### 2.6.3. NF-κB Pathway

The NF-κB pathway, a prototypical proinflammatory signaling pathway, has been observed to play a key role in cellular adaptation. As shown in Figure 4C, GA-T0 downregulates murine double minute-2 (MDM2), enhancing apoptosis [54] and cell cycle arrest at the G1 phase in SNB19 cells. This effect might involve the role of NF-κB targeting Bcl3 and NF-κB kinase subunit beta (*IKK2*) [55] by negatively regulating p53, thus suppressing NF-κB signaling. GA-T0-treated LN229 cells also showed upregulation of the NF-κB inhibitor-*α* (NFKBIA), which prompted our findings on the repression of the NF-κB pathway. The deletion or downregulation of NFKBIA is well associated with GBM progression and lack of response to therapies [56], in many types of cancers [57], suggesting the role of GA-T0 as a tumor suppressor. CNNB1, encoding β-catenin, was observed to be downregulated in SNB19, whose activation promotes proliferation, migration, and invasion in GBM [58] and oral squamous carcinoma [59].

#### 2.6.4. MAPK Pathway

Augmenting the effects of GA-T0 on the other pathways analyzed, inhibition of the genes related to the MAPK-dependent signaling pathways in both the GBM cell lines was also observed. Notably, *SPRY4*, coding for the sprouty 4 protein, was upregulated in LN229 cells, whose ectopic expression by GA-T0 inhibited the proliferation and migration of GBM cells. Its negative regulation of MAPK activation positions it as a tumor suppressor in GBM [60]. The expression of *STMN1*, coding for stathmin, was also found downregulated in both GBM cell lines, which might be due to the phosphorylation of Ser25 and Ser38 by MAPK [61,62] (Figure 4D). GA-T0 binding to the GPR17 receptor influences the downregulation of the cAMP level by decreasing the adenylyl cyclase activity, which in turn regulates various signaling pathways, such as the PI3K–Akt, Stat, NF-κB, and MAPK pathways. Thus, GPR17-mediated signaling activation promotes the inhibition of GBM tumor growth and proliferation (Figure 4E).

### 2.7. GA-T0 Crosses the Blood–Brain Barrier

**Figure 5:**
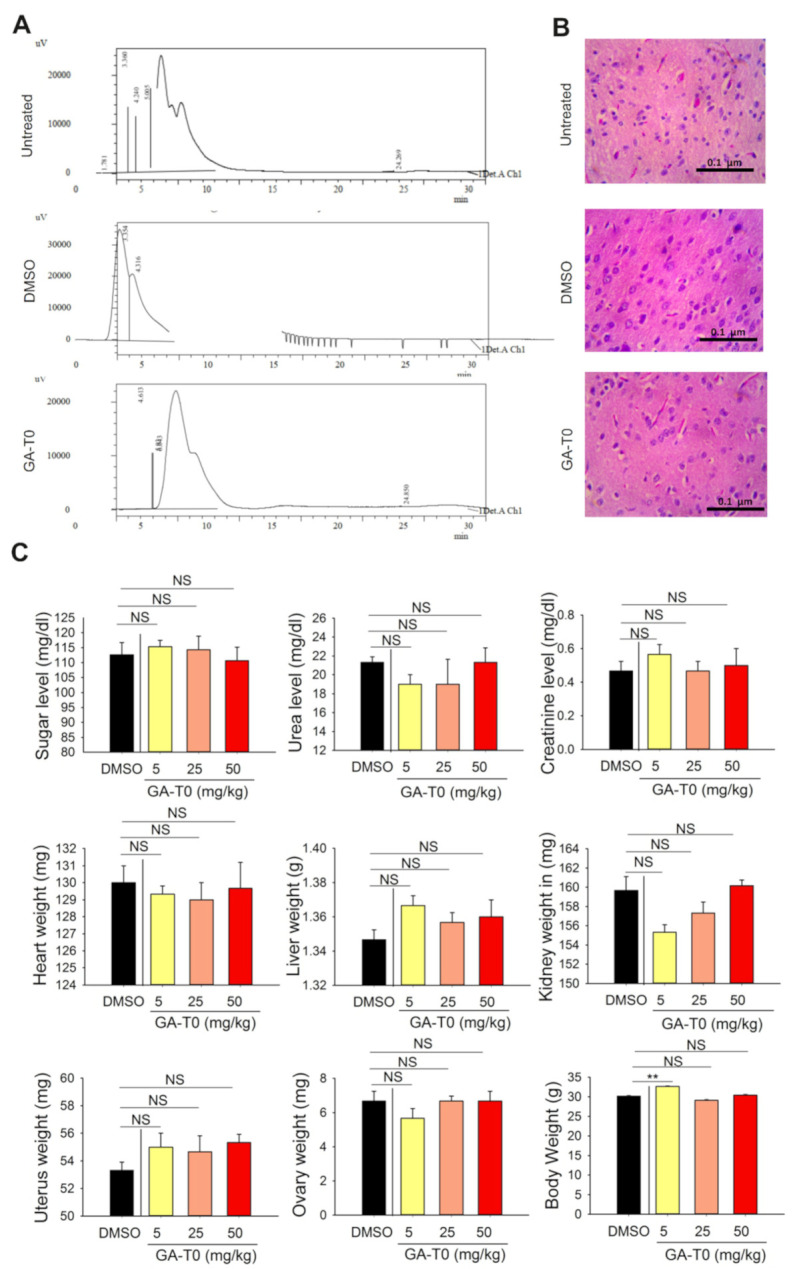
**Ability of GA-T0 to cross the BBB in an in vivo model.** (**A**) HPLC analysis showing the retention time in the control, DMSO, and GA-T0-treated *Mus musculus*. (**B**) Histopathology of the brain tissues from the control, DMSO, and GA-T0-treated wild mice. Photomicrographs of the cerebral cortex of the mice showing a normal architecture of the pyramidal neurons (PYC) in untreated and treated animals. (**C**) Changes in body weight, organ weight, and biochemical indicators, such as the sugar, creatinine, and urea (mg/dL) level, in wild mice upon GA-T0 treatment at varying concentrations, namely, 5, 25, and 50 mg/kg animal weight. Non-significant data are denoted by NS and significant data by asterisks; biological and technical repeats, *n* = 6, **, *p* < 0.01).

Being a strong agonist of GBM cell lines, causing potential cell death and cell cycle arrest, we further investigated the ability of GA-T0 to cross the blood–brain barrier (BBB) in wild mice, *Mus musculus*, using HPLC analysis. The retention time of GA-T0 was found to be 6.043, confirming it has the ability to cross the BBB in wild mice (Figure 5A). Histological analysis of the brain tissues showed no morphological or physiological changes in the brain cells (Figure 5B). Analysis of organ histology from GA-T0-treated mice identified no significant pathology in the weight (mg) of the heart, liver, kidney, ovary, and uterus. Assessment of biochemical nephrotoxicity indicators, such as sugar, creatinine, and urea (mg/dL), showed no significant differences compared to the controls, reflecting the ability of GA-T0 to maintain the metabolic homeostasis [63] of the extracellular environment (Figure 5C).

### 2.8. Preclinical Validation of GA-T0 in Patient-Derived Cell Lines (PDC) and Patient-Derived Xenograft Mouse Models (PDX)

**Figure 6:**
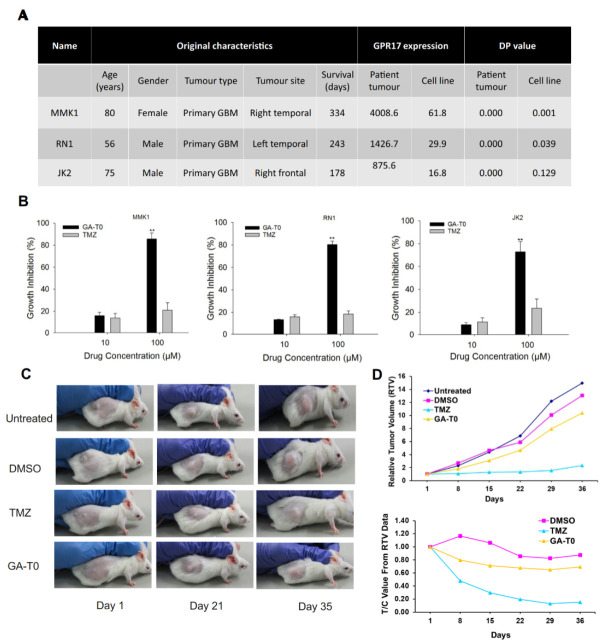
**The anti-tumor effect of GA-T0 on patient-tissue-derived GBM cells and PDX animal models.** (**A**) Demographic features of MMK1, RN1, and JK2 cells derived from GBM patients showing differential GPR17 expression. (**B**) Percentage of growth inhibition on patient-tissue-derived GBM cell lines upon treatment with 10 and 100 µM of GA-T0 and TMZ. (**C**) Images of xenograft GBM treated with DMSO (vector control), TMZ (positive control), and GA-T0 (Drug) at Day 1, Day 21, and Day 35. (**D**) Periodical validation of the relative tumor volume (RTV) and relative activity criteria (T/C) in PDX models on treatment with DMSO, TMZ, and GA-T0. (**A**) DP—detection *p*-value. Illumina beadchips allow a detection *p*-value to be calculated as an estimate of gene measurements relative to background. *p* < 0.05 for all samples on a beadchip for a given probe was used as a cut off to compile the gene expression datasets. (**B**) The results are presented as the mean values ± SEM of six experiments; significant data are denoted by asterisks (**, *p* < 0.01).

Preclinical validation was performed in patient-derived cell lines (PDC) and patient-derived xenograft mouse models (PDX). Stringer et al. (2019) cultured low-passage primary patient GBM cell lines, such as MMK1, RN1, and JK2, from different age groups, and their demographic features are represented in Figure 6A. Strikingly, microarray analysis revealed the expression variation of GPR17, where MMK1 was implicated as having the highest level of expression followed by RN1 and JK2. This is due to the heterogenous variation in gene expression exhibited in different GBM patients [64]. Inconsistent with our previous cytotoxicity results, there is no synergy between the action of TMZ in the patient-derived cell lines. Of note, there is less than 21% cell growth inhibition, even at a higher concentration of TMZ, whereas GA-T0 showed significant (*p* < 0.01) cell death of approximately 86%, 80%, and 73% in MMK1, RN1, and JK2, respectively, at a similar concentration (Figure 6B). There was a positive correlation between GPR17 expression and percentage of cell death in PDC-treated GA-T0 at 100 μM (r(9) = 0.680, *p* = 0.044) and 10 μM (r(9) = 0.777, *p* = 0.014) (Supplementary Figure S3). The response of the patients to the GPR17 agonist and TMZ treatment differs widely with host genetic variations and molecular background. The clinical diversity of the tumor cells also influenced its behavior to be distinct for the action of chemotherapy, TMZ.

To further support the potential clinical application of our results, we used patient-derived xenograft (PDX) mouse models generated from GBM cells. The animals were administered with GA-T0 and TMZ at a dose that exerted only a cytostatic effect (20 mg/kg). The relative tumor volume (RTV) and relative activity criteria (T/C) was periodically measured to validate the role of TMZ and the drug against the control and the vector control. We observed a sudden decrease in the tumor volume for GA-T0 and TMZ till the 8th day of treatment, with a substantial decrease till the 36th day of treatment (Figure 6C,D). The commercial chemotherapeutic agent (TMZ) exerts resistance to prolonged therapy with hematological toxicity [65], acute cardiomyopathy [66], oral ulceration, hepatotoxicity [67], and pneumocystis pneumonia [68], ultimately resulting in the discontinuation of therapy. The absence of GA-T0 toxicity in wild mice is considered more significant, whereas in PDX models, it is effective against tumor growth, and thus can potentiate progression-free survival through targeted GBM therapy.

## 3. Discussion

A glioblastoma possessing stable proliferation, invasion, and evasion of apoptosis, with increased angiogenesis, makes it susceptible to escape existing treatment strategies. The currently available drugs also focus only on either controlling inflammation or improving and modulating the patients’ immune response, and so no therapies and drugs have been found to provide protective activity against this disease. The potential target for GBM therapeutics has been improved by various comprehensive approaches to reduce its off-tumor toxicity; yet, it remains ambiguous. 

GPR17, an orphan G protein-coupled receptor, has been involved in oligodendrocyte differentiation, spinal cord injury, and brain injury [69]. Virtual high-throughput screening technology and in vivo assays identified galinex as a GPR17 agonist that significantly delays the onset of experimental autoimmune encephalomyelitis (EAE) [70]. In silico analysis revealed GPR17 upregulation in pediatric diffuse midline glioma clustering to *olig1* and *olig2* genes [26]. The proliferation rate of infratentorial LGG was controlled by various candidate genes, such as *ARX*, *GPR17*, *LHX2*, and *CXCL14*, where GPR17 is involved in the signal transduction pathway [71]. Our dataset analysis revealed constitutive expression of GPR17 in low-grade glioma (LGG) and GBM, where its expression is not only linked with improved survival but also significantly associated as a predictive biomarker.

Malignant gliomas, being lethal tumors, adjust to the environmental and genotoxic stress and thus promotes proliferation and invasiveness [72]. Our findings established the complex role of GPR17 signaling activation in the increased cytotoxicity against GBM cells, apoptosis, and thereby reduced cell proliferation. Gene expression analysis underscores the multifaceted role of GPR17 activation in the modulation of vital genes in several pathways, such as MAPK/ERK, PI3K–Akt, STAT, and NF-kB, controlling GBM disease progression. Additional in vivo data are also distinct, showing a reduction in tumor volume without affecting the local cellular environment, suggesting the potential role of GPR17 as a targeted therapy against GBM.

The extensive literature specifies the role of a renewed neurosphere in cultured glioma cells as a potential cause of patients’ death due to rapid tumor progression, involvement of proliferative genes, and signals from different pathways for the activation of G1/S phase [73,74,75,76]. Intriguingly, in vitro analysis revealed that activation of the GPR17 agonist favored the selective survival of Oligo 2 cells and altered the proliferative ability of glioma cells by decreasing the number of neurospheres [27]. Thus, neurosphere formation in GBM patients is considered as a significant predictor of clinical outcome, independent of tumor grade and patient age, and thus could reflect the clinical severity of glioma. Our work implicated that the PI3K–Akt pathway reduces the proliferation of the neurosphere on arresting the cell cycle at the G1 phase and decreases its number by increased apoptosis. The role of other pathways, such as the STAT, NF-κB, and MAPK pathways, in specifically regulating the neurogenic proliferation and their activation in GBM tumorigenesis remains to be elucidated.

Identifying the appropriate patient-specific treatment strategy is an unrelenting endeavor in GBM treatment. Lomustine, carmustine, temozolomide, and bevacizumab are the anti-GBM drugs approved by the FDA, out of which the former three drugs only have partial brain penetration, while bevacizumab fails clinical trials and does not show significant impact on patient survival [77,78,79]. In terms of GPR17-targeted therapy for GBM treatment, there are many novel compounds that are able to interact with the GPR17 receptor [80,81,82]. Unfortunately, there are no GPR17-targeted compounds under investigation in clinical trials. However, our data revealed that GPR17 signaling activation using GA-T0, observed to arrest cell cycle, induces apoptosis and show a cytotoxic effect against GBM cells, as well as in patient-derived cell lines, with significant tumor cytotoxicity in in vivo PDX animal models. Thus, abrogation of neural stem cell proliferation, myelin sheath damage, and infiltration to the nearby tissues through the sensor, such as GPR17 signaling activation, could benefit GBM treatment. Taken together, much remains to be discovered about the pharmacological mechanism of GPR17 receptor signaling for multiple subtypes of GBM, which opens the door for new hope in finding successful therapy for glioma treatment.

## 4. Materials and Methods 

### 4.1. Protein–Protein Docking and Docking Simulations

A comparative molecular interaction study was performed using the computational structured model of GPR17 [30] and X-ray crystallography structure of Guanine Nucleotide Binding Protein [alpha]I1 (GαI) [PDB. ID:1KJY, 2.70 Å] [83]. Cluspro, an FFT web-based docking server, was used to study the binding efficiency of these three interacting signaling proteins [84]. Simultaneously, High Ambiguity Driven protein–protein DOCKing (HADDOCK V.2.2) and *ab-initio* docking methods were also used to achieve the consensus scores [85]. Docking simulation was done for the earlier known GPR17 agonist, 2-carboxy-4,6-dichloro-1H-indole-3-propionic acid (MDL 29,951) [22] and T0510-3657(GA-T0), the recently identified novel agonist by our group via the Blind docking web server [86] (http://bio-hpc.ucam.edu/webBD/index.php/entry, accessed on 2 February 2018). For each ligand, 200 binding poses were generated and sorted based on the binding energy and conformation in the protein’s binding site. 

### 4.2. Cell Culture

SNB19 and LN229 human glioma cell lines (gifted by Dr.Kirsi Granberg, Faculty of Medicine and Health Technology, Tampere, Finland) and MEF, the mouse embryonic fibroblast cell line (gifted by Prof. Pasi Kallio, Faculty of Medicine and Health Technology, Tampere, Finland), were cultured in Dulbecco’s Modified Eagle Medium (DMEM) supplemented with 10% FBS, 0.1 mg/mL streptomycin, 100 U/mL penicillin, and 0.025 mg/mL amphotericin B (Sigma-Aldrich, St. Louis, MO, USA) under standard cell culture conditions (37 °C, 5% CO_2_). 

### 4.3. Expression Analysis of GPR17 at the mRNA and Protein Level in GBM Cells

Total RNA from LN229 and SNB19 cells was isolated using the GeneJET RNA Purification Kit (ThermoFisher Scientific, Waltham, MA, USA) following the manufacturer’s instruction. RNA was reverse transcribed using High-Capacity cDNA Reverse Transcription Kit (Applied Biosystems™, Waltham, MA, USA). The PCR was carried out to detect the expression of human GPR17 with primers described previously [20] (5′-GACTCCAGCCAAAGCATGAA-3′ and 5′-GGGTCTGCTGAGTCCTAAACA-3′). Housekeeping gene β--actin was used as an endogenous control (primers- 5′- CTGGGACGACATGGAGAAAA-3′ and 5′-AGGAAGGCTGGAAGAGTGC-3′) [87].

To further validate the expression of GPR17 in GBM cell lines at the protein level, an immunoblot assay was performed. For this, LN229 and SNB19 cells were lysed in ice-cold lysis buffer (25 mM Tris, pH 7.4, 150 mM NaCl, 1 mM EDTA, 1% Triton X-100, 1% IGEPAL), supplemented with protease inhibitor mixture (Sigma-Aldrich, St. Louis, MO, USA). The protein was separated by SDS-polyacrylamide gel electrophoresis and transferred to nitrocellulose membrane (Amersham^TM^ Protran^TM^ 0.45 µm NC, GE Healthcare Life Science). The membranes were blocked with BSA and stained with antibodies specific for GPR17 (1:500; sc-514723, Santa Cruz Biotechnology, Dallas, TX, USA) and α-Tubulin (1:1000; sc-8035, Santa Cruz Biotechnology). Signals were visualized using Odyssey CLx (LI-COR Biosciences, Lincoln, NE, USA) after staining the membranes with goat anti-mouse secondary antibody (1:5000; Dylight 800, Thermo Scientific). 

### 4.4. cAMP GloTM Assay

To evaluate the cAMP production in response to the effect of GPR17 agonist, GA-T0, cAMP GloTM Assay was performed. LN229 and SNB19 cells were seeded in a white 96-well plate (Nuclon, ThermoFisher Scientific, USA) at an initial density of 1 × 10^4^ cell/well. After overnight incubation, the cells were washed with PBS, incubated with 10 μM Forskolin (FK) (Sigma-Aldrich, St. Louis, MO, USA) for 15 min at 37 °C, and treated with 10 µM, 25 µM, 50 µM, 75 µM, and 100 µM of GA-T0 for 2 h. The cells were then harvested, lysed, and assayed for cAMP accumulation using the cAMP-GloTM Assay kit (Promega, Madison, WI, USA) following the manufacturer’s protocol. The luminescence intensity was measured using a Spark plate reader (Spark^®^, Tecan, Männedorf, Switzerland).

### 4.5. Measurement of the Intracellular Calcium Concentration

To determine the role of GPR17 in triggering the intracellular Ca^2+^, a Fura-2 AM assay was performed. GBM cells at 60–70% confluency were cultured in a black, clear bottom 96-well plate (Corning, Sigma-Aldrich), washed with PBS, and treated with 10 µM, 25 µM, 50 µM, 75 µM, and 100 µM of GA-T0. After 2 h of incubation at 37 °C, 100 µL Dulbecco’s PBS (Sigma-Aldrich, St. Louis, MO, USA) containing 5 µM Fura-2 AM (Sigma-Aldrich, St. Louis, MO, USA) and 0.1% Pluronic^®^ F-127 (Sigma-Aldrich, St Louis, MO, USA) was loaded into each well. Cells were incubated in darkness for 30 min and later washed twice with DPBS. The Ca^2+^ level was measured using a microplate reader (Spark^®^, Tecan) at two dual excitation/emission wavelengths of 340/510 and 380/310 [88]. The experiments were performed in triplicate for all the conditions. 

Similarly, the time-dependent effect of the Ca^2+^ level was also performed as described above, where the fluorescent signals were measured every 5 min in the microplate reader (Spark^®^, Tecan). The cells were treated with 50 μL of DPBS for treated condition and 100 μL of DPBS for untreated condition. At cycle 5 (after 20 min), 50 μL of GA-T0 (IC_50_) dissolved in DPBS was added to the treated condition and all the wells were subjected to the fluorescent measurement until it reached cycle 40. The experiment was performed with *n* = 6 in all the conditions and the fluorescent intensity was calculated using the following Equation (1).

340/380 ratio = (F_raw_ 340 − F_blank_ 340)/(F_raw_ 380 − F_blank_ 380) (1)

where Fraw 340 and Fraw 380 are the fluorescent intensities emitted at 510 nm between 340 nm and 380 nm excitation, respectively.

### 4.6. In Vitro Cell Proliferation Assay

The in vitro cytotoxicity activity of the GPR17 agonist, GA-T0, against SNB19, and LN229 cells was measured. The known GPR17 agonist, MDL 29,951, was used as the positive control and temozolomide (TMZ) as the drug control. An initial density of 1 × 10^5^ cells/well were grown in 12-well plates until 60–70% confluency and the cells were treated with a 10 µM and 100 µM concentration of the abovementioned compounds. The cells were incubated for 24 h in the controlled culture conditions and later centrifuged at 3000 rpm for 10 min. Live and dead cells were measured using trypan blue staining using Countless II FL Automated Cell Counter (ThermoFisher Scientific, Waltham, MA, USA). The percentage of inhibition of cell growth [89] was calculated using the following equation (2). Biological and technical replicates were conducted for each condition.

(2) $$Inhibition\;\left(\%\right)\;=\frac{Mean\;No.\;of\;untreated\;cells\;\left(control\right)\;-\;Mean\;No.\;of\;treated\;cells\times 100}{Mean\;No.\;of\;untreated\;cells\;\left(control\right)}$$

### 4.7. Pharmacodynamics Study

A pharmacodynamics study was performed to assess the effect of GA-T0 on the relationship between varying drug concentration and time course over cell growth. The study was performed as described previously for the in vitro cytotoxicity assay. The different concentration of GA-T0, 10 µM, 25 µM, 50 µM, 75 µM, and 100 µM was used to evaluate the cell viability on SNB19 and LN229 cells. The time-dependent study was performed for 24 h, 48 h, and 72 h exposure and a half maximal inhibitory concentration (IC_50_) was calculated from the dose–response curve. The calculated IC_50_ value at 24 h post treatment was used for further analysis. 

### 4.8. Apoptosis Annexin V-FITC/PI Apoptotic Assay

Quantitative assessment of apoptosis and necrosis for GA-T0 against SNB19 and LN229 cells was measured using a Dead Cell Apoptosis Kit using Annexin-V/fluorescein isothiocyanate (FITC) and propidium iodide (PI) (ThermoFisher Scientific, Waltham, MA, USA). Briefly, cells were seeded in 6-well plates at an initial density of 5 × 10^5^ cells/well. Cells were treated then with an IC_50_ concentration of GA-T0 for 24 h. Positive control (TMZ), negative control (DMSO), and untreated samples were also included in the experiment. The cells were collected, washed in ice cold PBS, and the cell pellets were resuspended in 1× annexin-binding buffer. To 100 μL of cell suspension, 5 μL of FITC conjugated annexin-V and 1 μL of the 100 μg/mL PI was added and incubated at RT for 15 min. Fluorescent images of the viable, apoptotic, or necrotic cells with differences in plasma membrane integrity and permeability were captured using an EVOS imaging system (ThermoFisher Scientific, Waltham, MA, USA). All the experiments were performed with *n* = 6 in all the experimental conditions. 

### 4.9. Cell Cycle Analysis by Propidium Iodide (PI)

The ability of GA-T0 to arrest cells at the G1 phase, S phase, and G2/M phase of the cell cycle was assessed using PI staining. SNB19 and LN229 cells were cultured in 6 well-plates at an initial density of 5 × 10^5^ cells/well and incubated overnight. The cells were treated with an IC_50_ concentration of GA-T0 and TMZ for 24 h, where DMSO was used as negative control along with the untreated samples. Cells were collected, washed in cold PBS, and fixed in 70% ice-cold ethanol for 30 min at 4 °C. The cells were then suspended in 200 μL PBS containing 20 µg/mL PI, 0.2 mg/mL RNase, and 0.1% triton X-100, and incubated for 30 min at 37 °C. Fluorescence images were captured by using an EVOS imaging system (ThermoFisher Scientific, Waltham, MA, USA) and cells arrested at different phases of the cell cycle analyzed using CellProlifer. 

### 4.10. Differential Gene Expression Analysis

High-throughput sequence-based Illumina RNA-seq was used to analyze transcripts for differential expression upon the drug treatment. Total RNA was extracted from GA-T0-treated LN229 and SNB19 cells at their respective IC_50_ concentration for 24 h, using the GeneJET RNA Purification Kit (ThermoFisher Scientific, Waltham, MA, USA). RNA sequencing was done by outsourcing in the Biomedicum Functional Genomics Unit (FuGU, University of Helsinki, Finland) using Illumina NextSeq 500 and the fold change in RNA expression was measured [15]. All the experiments were conducted in triplicates. 

### 4.11. Tumor Samples and Cytotoxicity Effect of GA-T0

The cytotoxicity effect of GA-T0 on patient-derived GBM cell lines, MMK1, RN1, and JK2 (gifted by QIMR Berghofer, Medical Research Institute, 300 Herston Rd, Herston QLD 4006, Australia) was analyzed. The isolation and development of cell lines from the patients were approved by the human ethics committee of the Queensland Institute of Medical Research and Royal Brisbane and Women’s Hospital [90]. The cells were cultured in serum-free conditional medium using 1% Matrigel-coated flasks in a humidified incubator at 37 °C supplied with 5% CO_2_ [91]. The cell lines were plated in 12-well plates with the initial density of 1 × 10^5^ cells per well and treated with 100 and 10 µM of GA-T0 and TMZ for 24 h. The cell growth inhibition was analyzed following the protocol described earlier.

### 4.12. In Vivo Experiments

#### 4.12.1. Wild Mice

All protocols involving normal mice, *Mus musculus*, were approved by the Institutional animal ethics committee (IAEC) of the department of Animal science at Bharathidasan University, Tiruchirappalli, Tamil Nadu, India (Reg.No:418/GO/Re/S/01/CPCSEA, dt.24.07.2018). Adult female mice weighing 20–25 g were maintained in controlled environmental conditions, including a temperature of 25 ± 2 °C with 12 h dark/light cycle, a standard laboratory diet, and water ad libitum. Grouping of animals (*n* = 5/group) was done as follows: Treated Groups A, B, and C (GA-T0 with 5 mg, 25 mg, and 50 mg/kg); Group D (vehicle control, 0.1 mL of DMSO/kg); and Group E (control, untreated). The body weight of the animal was recorded periodically at Days 0, 7, and 15. All the mice were immobilized, sacrificed for the recovery of organs (lungs, heart, kidney, ovary, and uterus), and their weight noted before subjecting to further histopathology analysis.

#### 4.12.2. Histopathology Analysis of the Brain Tissues

The brain tissues were dissected after treatment and fixed in Bouin fixative solution for 24 h, processed in ethanol and embedded in paraffin. Microtome sections 5 µm thick were stained with hematoxylin and eosin and viewed under a light microscope (Olympus BX51, Tokyo, Japan) for any morphological and physiological changes. HPLC was performed with a Shimadzu (model UFLC) HPLC apparatus equipped with a UV-visible detector (235 nm) and Shim-pack GIST-HP C18 column, with an acetonitrile, pH 7.4, phosphate buffer 1:1 (*v*/*v*) and flow rate of 1.5 mL/min. All compounds were injected as 0.1 mg/mL solutions in DMSO (injection volume–20 μL). All chromatograms were repeated (*n* = 6), and the mean k values were used for further investigations.

#### 4.12.3. Patient-Derived Xenografts (PDXs)

In vivo cancer activity was evaluated against glioblastoma U373-MG Uppsala (https://web.expasy.org/cellosaurus/CVCL_2818, accessed on 24 June 2021) in human tumor xenograft mouse models. All protocols were approved by the Institutional Animal Ethics Committee, ACTREC, Tata Memorial Centre, Navi Mumbai (Ethical number: 01/2015), and adhered to CPCSEA guidelines (Registration Number: 65/GO/ReBiBt/S/99/CPCSEA). In-house bred Balb/c or NOD-SCID mice of six to eight weeks old were used in the experiments. Animals were maintained with utmost human care and all measures were taken to minimize animal suffering before and during the experiments.

**Acute toxicity studies**

Acute toxicity for GA-T0 by intraperitoneal route was determined using six immunocompetent Balb/c mice per dose. Mortality and weight loss ≥4 g/mouse were considered as the toxicity criteria. The dosage of the drug given was 20 mg/kg body weight of the animal and was injected every 7 days for 30 days. The mice were monitored for any of physical sign of morbidity or mortality after 5 days post-dosing of the drug.

**Experimental design**

All the mice were randomized into the desired experimental groups (*n* = 6/group). The experimental group comprised of the control (Group A); vehicle control—DMSO (Group B); positive control—temozolomide (Group C); and GA-T0 (Group D). Tumor measurements were carried out to determine the tumor growth and tumor volume using digital Vernier calipers (Pro-Max, Electronic Digital Caliper, Fowler-NSK, USA). Mice were observed at regular intervals for a period of around 36 days for various features, such as the body weight, tumor volume, and mortality.

**Statistical calculation for in vivo studies**

The data are represented as the relative tumor volume in cubic centimeters (RTV in c.c), T/C (ratio of test versus control), and survival. Tumor volume was calculated using the formula ((w1 × w1 × w2) × (π/6)), where w1 and w2 were the smallest and the largest tumor diameter (cm), respectively. RTV was measured as tumor volume on the day of measurement/tumor volume on Day 1. The T/C ratio indicates antitumor effectiveness. The percentage treatment/control (T/C %) values or percent tumor regression values were calculated using the following equation:

(RTV)T/C = RTV_Test/RTV_Control Tumor Regression % = 100 − [T/C * 100](3)

where T = mean tumor volume of the drug-treated group; RTV = mean tumor volume of the drug-treated group on the study day of interest—mean tumor volume of the drug-treated group on the initial day of dosing; and C = mean tumor volume of the control group. As per NCI, USA guidelines, biological activity was considered significant when T/C values were ≤0.42.

## 5. Conclusions

GPR17 expression is associated with higher survival for both low-grade glioma (LGG) and glioblastoma (GBM). GA-T0, a potent GPR17 receptor agonist, causes significant GBM cell death and apoptosis. Upregulation of *DDIT3*, *DDIT4*, and *SQSTM1* genes showed a significant role in inducing GPR17-activated cell damage. Apoptotic inhibitor genes, such as survivin, *BIRC5*, and *API5*, were downregulated with the upregulation of the proapoptotic genes, such as *BBC3* in SNb19. Downregulation of *CASP2*, *CASP3*, and *CASP7* reveals the GPR17-mediated, caspase-independent mechanism of apoptosis. GPR17 signaling promotes cell cycle arrest at the G1 phase in GBM cells. Key genes are modulated in the signaling pathways such as the MAPK/ERK, PI3K–Akt, STAT, and NF-κB pathways, which inhibit GBM cell proliferation. GA-T0 crosses the blood–brain barrier and reduces tumor volume in the xenograft model. These results suggest that targeting the GPR17 receptor presents a novel therapeutic target for the treatment of glioblastoma.

## Data Availability

The data are available from the corresponding authors upon request.

## Supplementary Materials

The following are available online at https://www.mdpi.com/article/10.3390/cancers13153773/s1, Figure S1: GPR17 expression profile across all tumor samples and paired normal tissues. Dots represent expression in individual samples. Figure S2: Two-dimensional interaction diagram for MDL 29,951-protein complex. Figure S3: Correlation between GPR17 expression and percentage of cell death in three Patient derived cell lines treated with 100 µM and 10 µM of GA-T0. Spearman’s (p) and Pearson’s (r) correlation between the two values are shown. Figure S4: Western blot analysis of GPR17 receptor protein expression in LN229 and SNB19 cells. Table S1: The list shows the genes that were differentially expressed in GA-T0 vs Untreated in the cell type analysis using DESeq2. Table S2: The list shows the genes that were differentially expressed in GA-T0 vs Untreated in the cell type analysis using DESeq2.

## Abbreviations

GBM	Glioblastoma multiforme
GPCR	G protein-coupled receptor
GA-T0	GPR17 agonist T0510-3657
pDMG	Pediatric diffuse midline glioma
CysLT	Cysteinyl-leukotriene
TMZ	Temozolomide
ROS	Reactive Oxygen Species
RNA-seq	RNA-sequencing
DMEM	Dulbecco’s Modified Eagle’s Medium
DNA	Deoxyribonucleic acid
FBS	Fetal Bovine Serum
DEG	Differentially expressed gene
