# Supplementary material for: Targeting Orphan G Protein-Coupled Receptor 17 with T0 Ligand Impairs Glioblastoma Growth
Source: Cancers (Basel). 2021 Jul 27;13(15):3773. doi: 10.3390/cancers13153773 (PMC8345100; doi:10.3390/cancers13153773)
Supplement: Supplementary file 1 [file cancers-13-03773-s001.zip › cancers-1290797 supplementary.pdf]

Targeting orphan G Protein–Coupled Receptor 17 with T0 ligand impairs glioblastoma growth

Phuong Doan [1,2,3#], Phung Nguyen [1,2,3#], Akshaya Murugesan[1,2,3], Kumar Subramanian[1,2,3], Saravanan Konda Mani[4], Vignesh Kalimuthu[5], Bobin George Abraham[6], Brett Stringer[7], Kadalmani Balamuthu[5], Olli Yli-Harja[8,9] and Meenakshisundaram Kandhavelu[1,2,9,*]

[1] Molecular Signaling Lab, Faculty of Medicine and Health Technology, Tampere University, P.O. Box 553, 33101 Tampere, Finland
[2] BioMediTech Institute and Faculty of Medicine and Health Technology, Tampere University, Arvo Ylpön katu 34, 33520 Tampere, Finland
[3] Department of Biotechnology, Lady Doak College, Thallakulam, Madurai – 625002, India
[4] Shenzhen Institutes of Advanced Technology, Chinese Academy of Sciences, Shenzhen, Guangdong, China
[5] Department of Animal Science, Bharathidasan University, Tiruchirappalli-620024, India
[6] Faculty of Medicine and Health Technology, Tampere University, P.O. Box 553, 33101 Tampere, Finland
[7] College of Medicine and Public Health, Flinders University, Sturt Rd, Bedford Park South, 5042, Australia
[8] Computational Systems Biology Group, Faculty of Medicine and Health Technology, Tampere University, P.O. Box 553, 33101 Tampere, Finland
[9] Institute for Systems Biology, 1441N 34th Street, Seattle, WA, USA
* Correspondence: meenakshisundaram.kandhavelu@tuni.fi; Tel.: (+358)417488772
\# Equal Contribution

Supplementary Figure 1

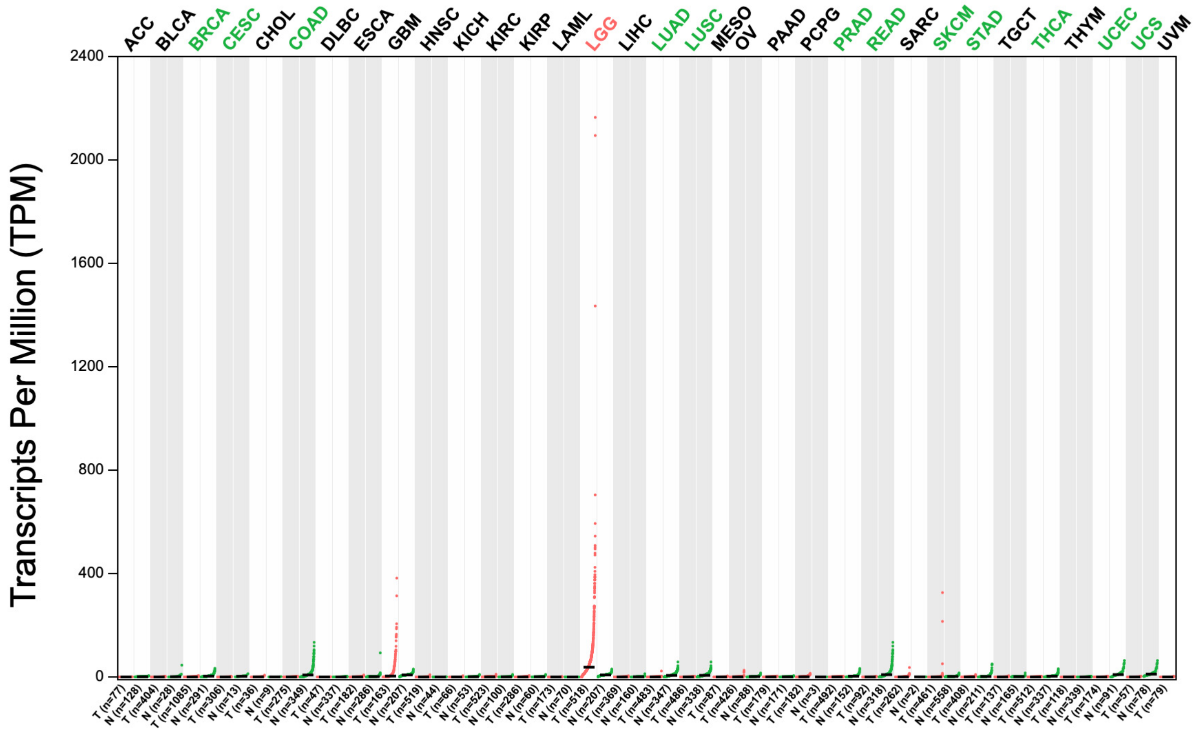

Supplementary Figure 1: *GPR17* expression profile across all tumor samples and paired normal tissues. Dots represent expression in individual samples.

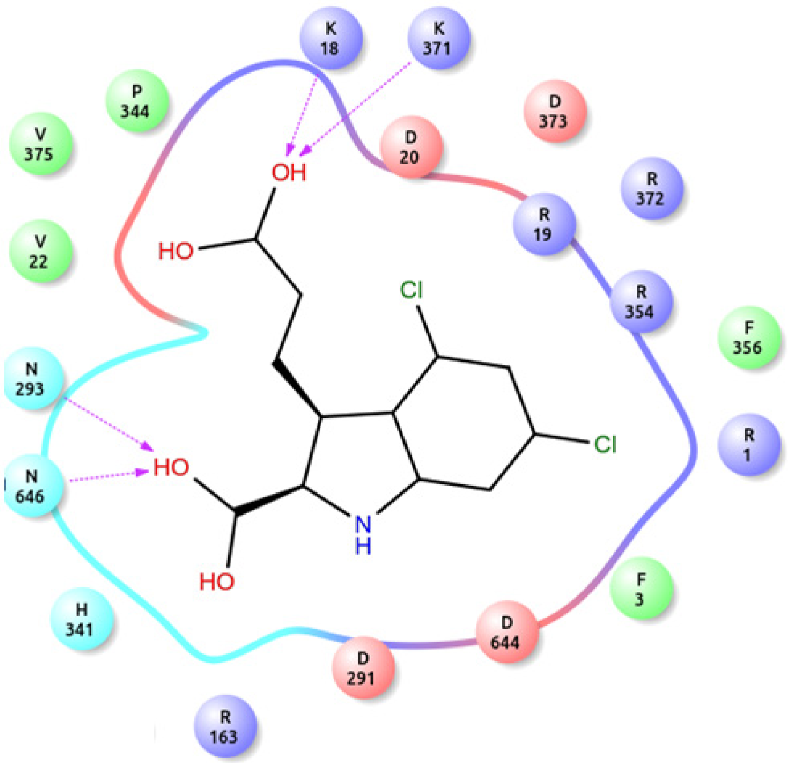

Supplementary Figure 2. Two-dimensional interaction diagram for MDL 29,951-protein complex.

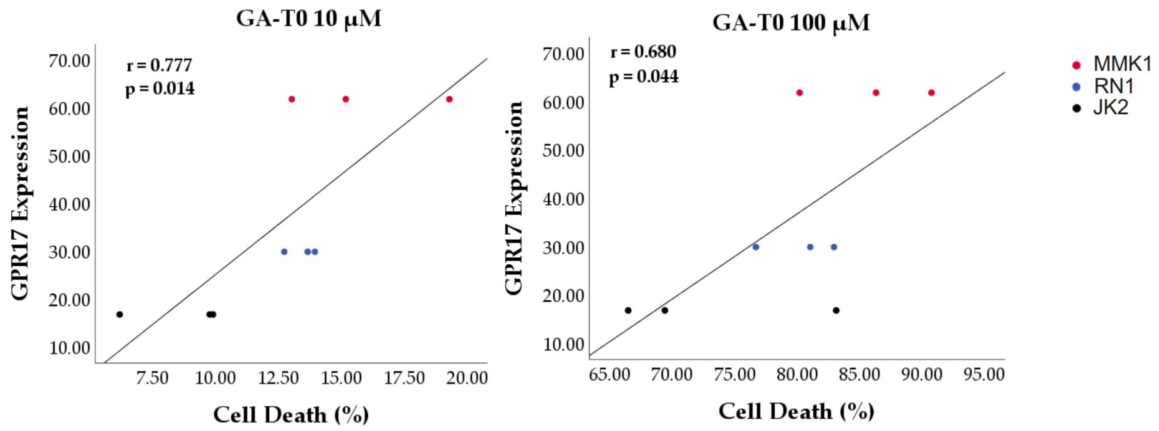

Supplementary Figure 3**.** Correlation between GPR17 expression and percentage of cell death in three Patient derived cell lines treated with 100 µM and 10 µM of GA-T0. Spearman's (p) and Pearson's (r) correlation between the two values are shown.

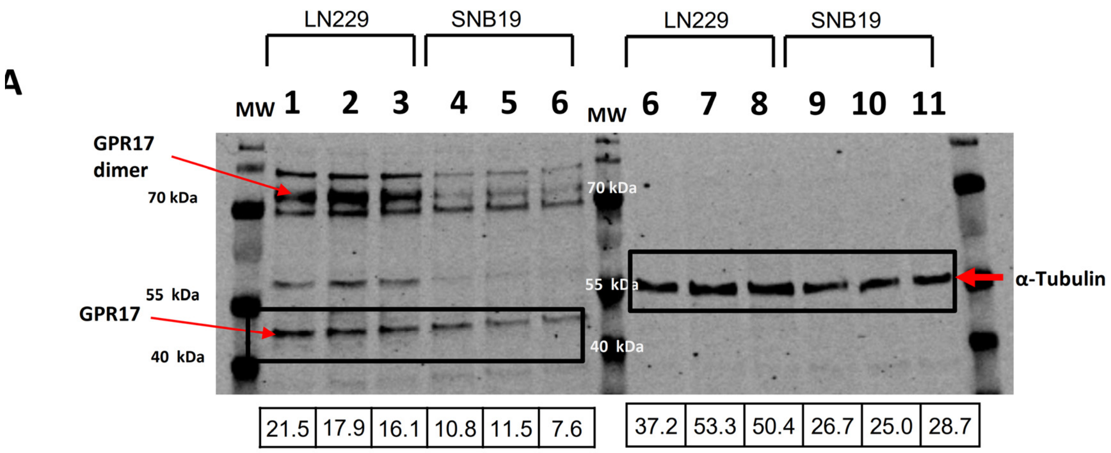

Signal / intensity ratio from the above western blot

	LN229			SNB19		
GPR17	21.5	17.9	16.1	10.8	11.5	7.6
α-tubulin/ GPR17	1.7	3.0	3.1	2.5	2.2	3.8

LN229			SNB19			
37.2	53.3	50.4	26.7	25.0	28.7	α-tubulin

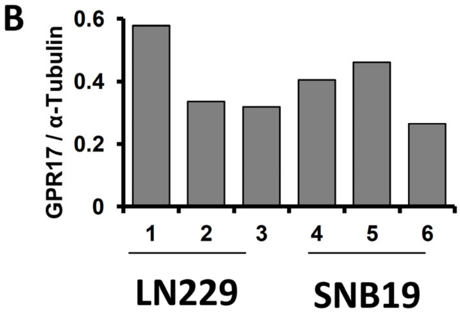

Supplementary Figure 4: A) Western blot analysis of GPR17 receptor protein expression in LN229 and SNB19 cells. MW: Molecular weight standard; Lane 1-3: LN229 triplicates; Lane 4-6: SNB19 triplicates; α-Tubulin as a loading control: Lane 6-8 for LN229 triplicates and Lane 9-11for SNB19 triplicates. The figure also shows the densitometry readings/intensity ratio of each band. B) Histogram showing the densitometric analysis of GPR17/α-Tubulin for LN229 (1-3) and SNB19 (4-6) cells.
